# Gonial Angle by Lateral Cephalogram in Orthodontic Patients of a Tertiary Care Hospital: A Descriptive Cross-sectional Study

**DOI:** 10.31729/jnma.6482

**Published:** 2021-05-31

**Authors:** Manju Bajracharya, Anjana Rajbhandari, Resina Pradhan, Pushkar Manandhar, Surendra Maharjan, Bashu Dev Pant

**Affiliations:** 1 Department of Orthodontics, People's Dental College and Hospital, Sorhkhutte, Kathmandu, Nepal

**Keywords:** *craniofacial*, *orthodontic*, *radiographs*

## Abstract

**Introduction:**

The Gonial angle is an important parameter of the craniofacial complex for growth Patterns prediction. The gonial angle on lateral cephalometric radiograph represents the mandibular morphology concerning mandibular body and ramus. The objective of this study was to find out the mean value of gonial angle in lateral cephalometric radiographs of patients of orthodontic department in a tertiary care center.

**Methods:**

The descriptive cross-sectional study was conducted among patients from the Department of Orthodontics at People's Dental College and Hospital between 8th December 2020 to 8th February 2021 at People's Dental College and Hospital, Kathmandu, Nepal after obtaining Ethical approval (Reference Number. 01, CH100 09,2077/2078) by the Institutional Review Committee. A convenience sampling technique was used to collect 166 pre-treatment lateral cephalograms radiographs of patients between 17-30 years. Data were collected and entered using Statistical Package of Social Science 16.

**Results:**

The mean value of gonial angle on lateral cephalogram radiographs was 132.84±3.70 in hyperdivergent, 119.94±5.57 in hypodivergent and 124.06±3.88 in normodivergent vertical skeletal patterns and between male and female were 132.52±4.32, 133.07±3.28 in hyperdivergent, 121.46±3.78, 119.14±6.42 in hypodivergent and 123.74±5.14, 123.94±3.90 in normodivergent vertical skeletal patterns of Orthodontic Patients.

**Conclusions:**

The gonial angle value on lateral cephalometric radiographs was greater in hyperdivergent than hypodivergent and normodivergent vertical skeletal patterns. The hyperdivergent vertical skeletal pattern of female was greater than of male patient's lateral cephalometric radiographs while hypodivergent and normodivergent vertical skeletal patterns of males were greater than female lateral cephalogram radiographs of Nepalese orthodontic patients.

## INTRODUCTION

The gonial angle on lateral cephalometric radiograph represents the mandibular morphology concerning mandibular body and ramus which is an important parameter for growth prediction pattern, rotation of the mandible.^1^ The high mandibular plane angle group showed increased gonial angle and low mandibular plane angle showed decreased gonial angle in lateral cephalograms.^2^ Gonial angle is significant for diagnosis of craniofacial disorder. Gonial angle is significantly increased in hyperdivergent group with no sexual dimorphism.^3^

The gonial angle value on the lateral cephalometric radiographs among different vertical skeletal types will be helpful for orthodontist in diagnosis and treatment planning. Various studies have been done for the study of gonial angle in lateral Cephalograms among different vertical skeletal patterns, but no studies have been done among different vertical skeletal patterns of Nepalese orthodontic patients.

This study aimed to find out the mean of gonial angle values on lateral cephalogram radiographs among three vertical skeletal patterns of patients of orthodontic department in a tertiary care center.

## METHODS

A descriptive cross-sectional study conducted from 8th Dec 2020 to 8th Feb 2021 at People's Dental College and Hospital, Nayabazar, Kathmandu, Nepal. Ethical clearance (Reference No. 01, CH100 09,2077/2078) was obtained from the Institutional Review Committee of Peoples Dental College and Hospital, Kathmandu, Nepal. A written and signed consent form was taken from the patients about radiation hazards before pre-treatment radiographic record collection. A convenience sampling technique was used to collect a total of 166 samples of pre-treatment Lateral cephalograms of 69 male and 97 female patients from the department of orthodontics at People's Dental College and Hospital with age ranging between 17-30 years. All the clinical records of the patients were verified and reviewed by the principal investigator. All the pre-treatment patient's radiographs with clear landmarks between the age of 17-30 years were included. The patient's radiographs having history of trauma, craniofacial abnormalities, orthodontic treatment before and having incomplete data were excluded from the study.

The sample size was calculated by using the formula,


$$\begin{array}{ll} \text{n} & \text{= }{\text{Z}}^{\text{2}}\times {\text{σ}}^{\text{2}}\;/{\text{e}}^{\text{2}}\\ & \text{= }{\left(1.96\right)}^{\text{2}}\times {\left(6.59\right)}^{2}/{(\text{1)}}^{\text{2}}\\ & \text{= }166 \end{array}$$

Where

Z = 1.96 at 95% confidence level
σ = standard deviation (6.59) according to reference article Pokharel M, et al. in 2019^9^
e = margin of error, 1

The records were further divided into three groups according to their vertical skeletal patterns, depending upon the angle formed by a mandibular plane that is (Go-Me) to Frankfort horizontal plane (F.H) on lateral Cephalogram. The mean value of mandibular plane angle was taken as according to Rajbhandari, et al. In hypodivergent vertical skeletal pattern is <23.2 degrees, normodivergent mean value of mandibular plane angle >23.2° to <32°, hyperdivegent of mandibular plane angle is ≥32°.^8^

Lateral cephalometric radiographs were obtained with the teeth in centric occlusion using a Cephalostat. Images were obtained using Sirona Orthophos SL. The patients were exposed at 73kV-15mA to 84kV-13mA, with standard procedures and natural head position. In the selected 166 samples, the name was blinded on the radiographs. All the lateral Cephalograms radiographs were traced manually on acetate tracing paper with a 3H sharp pencil on a view box using transilluminated light in a dark room. The mathematical protractor and standard metallic scale were used to measure the linear measurement of the gonial angle. The gonial angle was measured from the angle formed by the line joining Gonion (Go) to Menton (Me) and condylon (Co) to Gonion (Go) according to William B. Down's analysis.^4,6^

The Gonial angle was measured by the intersection of tangents drawn to lower border of mandible and tangent drawn to the posterior border of condyle and ramus (degree).^7^

All the information gathered was recorded in proforma which consists of demographic data (date, gender, age), mandibular plane angle according to down's analysis, angle in cepahlograms, and data was entered into Microsoft excel.

Systematic error in this study has been reduced by using lateral cephalograms radiograph from the same x-ray machine by a single observer. Fifty lateral cephalograms radiograph were selected randomly by lottery basis and were re-measured in one-week interval time for the intra-observer variation test. Cronbach Alpha was used for intra-observer variation test for lateral cephalogram radiographs which were observed to be 0.997 which was indicated more reliability between the measurement. The obtained data were entered using Statistical Package of Social Science (SPSS) version 16.

## RESULTS

The mean angle on lateral cephalogram radiographs was 132.84±3.70 in hyperdivergent skeletal patterns, 119.94±5.57 in hypodivergent skeletal pattern and 124.06±3.88 in normodivergent skeletal pattern (Table 1).

**Table 1:** Gonial angle on lateral cephalogram among different vertical skeletal patterns.

Vertical Skeletal patterns	Gonial angle on Lateral cephalograms
	Mean ±SD
Hyperdivergent	132.84±3.70
Hypodivergent	119.94±5.57
Normodivergent	124.06±3.88

The study group consisted of 166 pre-treatment patient's radiographs. The subjects were divided into three groups according to vertical skeletal patterns in relation to mandibular plane with Frankfort horizontal plane, that is, hyperdivergent-49 (29.5%) subjects, normodivergent-54 (32.5%) subjects, hypodivergent-63 (38%) (Table 2).

**Table 2:** Number of patients and their frequency (n = 166).

Characteristics	n (%)
**Sex**	
Female	97 (58.4)
Male	69 (41.6)
Total	166 (100)
Mandibular vertical skeletal pattern	
Hyperdivergent	49 (29.5)
Hypodivergent	63 (38)
Normodivergent	54 (32.5)
Total	166 (100)

The mean gonial angle on lateral cephalogram radiographs between male and female were 132.52±4.32 and 133.07±3.28 in hyperdivergent vertical skeletal patterns, 121.46±3.78, 119.14±6.42 in hypodivergent vertical skeletal pattern and 123.74±5.14,123.94±3.90 in normodivergent vertical skeletal pattern (Table 3).

**Table 3:** Mean and Standard Deviation of male and female among different vertical skeletal patterns.

**Vertical skeletal patterns**	**Male**		**Female**	
	**n**	**Mean±SD (degree)**		**n**		**Mean±SD (degree)**	
**Hyperdivergent**	22	132.54±4.21	27	133.07±3.28
**Hypodivergent**	28	120.93±4.17	35	119.14±6.42
**Normodivergent**	20	124.25±3.92	34	123.94±3.90

## DISCUSSION

The descriptive cross-sectional study of Gonial angle was performed on lateral cephalogram radiographs among different vertical skeletal (hyperodivergent, normodivergent, hypodivergent) of Nepalese orthodontic patients of a Tertiary Care Hospital. The Lateral Cephalograms are routinely taken for every orthodontic patient for diagnosis purpose. The goal of Cephalometric analysis is to evaluate the horizontal and vertical relationships of the cranium and cranial base, skeletal maxilla, skeletal mandible, the maxillary dentition and alveolar process and the mandibular dentition and alveolar process. The vertical relationships are as important as horizontal relationships for treatment planning.^10^ Orthodontist often used angle as an predictor of growth pattern of mandible. The patients with downward and backward rotation of mandible showed increased in angle value in hyperdivergent vertical skeletal patterns while upward and forward rotation of mandible showed decreased in angle value in hypodivergent vertical skeletal patterns.^2,5^ The mandibular plane angle was found to be most reliable indicators for assessing the vertical growth pattern of face.^11^

The result of the present study demostrated that the angle value in hyperdivergent vertical skeletal pattern was greater than hypodivergent and normodivergent vertical skeletal patterns of orthodontic patients which agrees with the previous studies done by Akcam MO, et al. Xiao D, et al. Mangla R, et al. Sharma VK, et al. Zhao Z, et al.^1-3,13,16^

The descriptive statistic for gender revealed the mean value of Gonial angle on lateral cephalogram radiographs was greater in female than male hyperdivergent vertical skeletal patterns (132.54±4.21, 133.07±3.28) while the mean value was greater in male compare to female hypodivergent vertical skeletal pattern (120.93±4.17, 119.14±6.42) and in normodivergent vertical skeletal pattern (124.25±3.92, 123.94±3.90). This study was disagreement with the study done in young Lebanese population who demonstrated no sex difference of the mandibular angle using lateral cephalogram radiographs.^12^ Again, this studies agrees the study done by Larrazabal-Moron C where males aged over 16 years old presented significantly higher angle values than the females of young Caucasian.^14^

Another study done by Bulut O, et al. who reported that males showed slightly smaller angle values than those of females in all age groups which agrees with the present study in hyperdivergent vertical skeletal pattern patients between 17-30 years of age but disagrees this study interms of hypodivergent and normodivergent patients which shows greater angle value in male compare to female patients.^15^

This study is two-dimensional studies of three dimensional structure of angle in lateral cephalogram which shows error in image magnification, superimposition, landmark identification, tracing, and measurements so the result of the study cannot be generalized. The present study is conducted in small sample size of lateral cephalogram radiographs, so further study with large sample size and digital radiographs is recommended.

## CONCLUSIONS

The mean value of gonial angle in hyperdivergent was greater than hypodivergent and normodivergent vertical skeletal patterns of orthodontic patients. The mean value of gonial angle on lateral cephalogram radiographs was greater in female than male in hyperdivergent vertical skeletal pattern but the mean value of gonial angle on lateral cephalogram was greater in male compare to female in hypodivergent and normodivergent vertical skeletal patterns of patients of orthodontic department in a tertiary care center. The gonial angle value on the lateral cephalometric radiographs among different vertical skeletal patterns will be helpful for orthodontist in diagnosis and treatment planning.
